# A revised prosocial behavior game: Testing associations with psychopathic traits and the effects of moral elevation using a randomized clinical trial

**DOI:** 10.1371/journal.pone.0283279

**Published:** 2023-04-19

**Authors:** Joseph T. Sakai, Yaswanth Chintaluru, Kristen M. Raymond, Shannon McWilliams, R. McKell Carter, Drew E. Winters, Susan K. Mikulich-Gilbertson

**Affiliations:** 1 University of Colorado School of Medicine, Anschutz Medical Campus, Aurora, Colorado, United States of America; 2 University of Colorado Boulder, Boulder, Colorado, United States of America; Juntendo University, JAPAN

## Abstract

**Background:**

Prosocial behavior is negatively associated with psychopathic traits and paradigms which measure prosocial behavior in the laboratory may be useful in better understanding moderators of this association.

**Methods:**

We revised a previously validated game of prosocial behavior by including a new trial type (i.e., trials where the participant will lose money and the charity will gain money). This version of the game was administered online and participants were randomized to group (exposed to a control stimulus video or a video used to elicit moral elevation, i.e. a positive response to witnessing another’s act of kindness). We used repeated game administration to test whether a moral elevation stimulus affected game behavior and moderated the negative association between psychopathic traits and prosocial behavior.

**Results:**

Prosocial behavior on the new trial types added in this revised game correlated strongly with prosocial behavior on the old trial type (i.e., trials where the participant will gain money and the charity will lose money; *r* = 0.71; *p-value*<0.001; n = 485). Graphing trial acceptance rates by trial characteristics demonstrated expected patterns of behavior. Number of prosocial choices on the game correlated with psychopathic trait score (Levenson Factor 1 score; *r* = -0.52; *p-value*<0.001). Game repetition with a control stimulus in between runs, supported high immediate test-retest reliability of overall game behavior. Exposure to the moral elevation stimulus in between runs did not affect game behavior nor moderate the association between psychopathic traits and prosocial behavior.

**Conclusions:**

Choices on this revised game of prosocial behavior, which can be administered online, are associated with psychopathic traits scores. The game appears to have high immediate test-retest reliability. Exposure to the moral elevation stimulus did not affect prosocial behavior or impact the relationship between psychopathic trait scores and prosocial behavior. Future research should continue to test potential moderators of this relationship. Limitations of the current study are discussed.

## 1. Introduction

Prosocial behavior, “behavior which the actor expects will benefit the person or persons to whom it is directed” [1] and altruism, a “moral call to place the needs of others over one’s self-interests” [2] have long been areas of interest in psychology and sociology [3]. Humans often engage in prosocial behaviors [4], even sometimes helping others when there is a cost to themselves [4].

Prior studies have utilized multiple approaches to measure prosocial behavior. These include self-report, categorical measures (e.g., offered the opportunity to volunteer or donate–with outcomes being yes/no), economic games, which generally involve interactions between two or more participants [5–7] and various other lab-based tasks [8–14]. Paradigms testing prosocial behavior can be categorized based on several factors including: the number of players, the characteristics of the other/beneficiary (e.g., real person vs. charity), the identity of the other (e.g., anonymous vs. a good actor), number of interactions (e.g., single decision game), whether the beneficiary will also make decisions and the probability and magnitude of the outcomes (see [15] for a review). One single-player game, the AlAn’s (**Al**triusm-**An**tisocial) game, presents a series of offers which require weighing self-interest and other harm [16]. The game provides quantitative outcomes, assesses behavior (instead of self-report) at differing levels of relative self-benefit, involves no opportunities for reciprocity, is devoid of assessment of risk (given some patient populations assess risk differently) and players are told that no information regarding how they played the game will be shared with others (reducing concerns about reputation) [16–18]. However, the AlAn’s game was developed to examine antisocial behavior phenotypes and the game trials (Trial Type A) ask players to accept/reject offers that will harm another (reduction in the charity donation) and benefit themselves (monetary gain to self). Greater number of accepted trials (Trial Type A) have been associated with higher levels of callous unemotional traits in adolescents [16, 18], greater levels of psychopathic traits in young adults [17] and are negatively associated with empathy [19].

While declining Trial Type A has generally been considered a prosocial act in prior papers [16, 17], prosocial and altruistic behaviors are often instead defined by actively helping another at cost to ones-self. In this revised game, new game trials (Trial Type B) were added, which ask players to accept/reject offers, which will help another (increase the charity donation) at a cost to themselves (monetary loss). We ask here, whether these new trials (Trial Type B) are considered differently by subjects when compared with the original trials (Trial type A). We sought to examine the pattern of behavior on these newly included trials and to address these specific questions: is prosocial behavior on these new trials associated with behavior on the old trials? Is the pattern of behavior observed logical? And does behavior on this revised game overall associate with psychopathic trait scores?

There has been increasing interest in studying approaches to enhancement of prosocial behavior. Prosocial behavior has been associated with emotional well-being, life satisfaction and happiness [20, 21], appears to be protective against several negative mental health outcomes [22–24] and successful experimental manipulation to increase prosocial behavior leads to increased happiness [25]. Prior work supports that positive emotional states may be critical for promoting and rewarding prosocial behaviors [26]. One positive emotional state, which can be induced in the laboratory through stimulus stories [27] or videos [17] is moral elevation [28, 29]. Witnessing acts of moral beauty (e.g., highly giving acts) elicits both emotional [29] and physical changes [30], with participants reporting a sense of being ‘uplifted’, ‘elevated’, or ‘moved’, by a “virtuous display” [5]; moral elevation has been shown in a handful of studies to motivate prosocial actions [7] e.g., as measured by volunteering for an unpaid study [31], helping the experimenter with a tedious task [31] and showing more giving behavior on the dictator game [6]. Here we sought to test experimentally whether participants exposed to a moral elevation stimulus increased their prosocial behavior on the AlAn’s game more than participants exposed to a neutral control stimulus.

In addition, we further sought to test the hypothesis that exposure to a moral elevation stimulus, at least in part, moderates the negative relationship between psychopathic traits and prosocial behavior in comparison to exposure to a neutral stimulus. Individuals with high levels of callousness and psychopathic traits have a blunted self-reported moral elevation response [16, 17], and exhibit less prosocial behavior [17]. Exposure to moral elevation stimuli is associated with increased prosocial behavior [6, 7]. Two prior studies using a cross-sectional design, have provided preliminary results suggesting that differences in the experience of moral elevation may in part account for the relationship between antisocial phenotypes and prosocial behavior [16, 17]. This randomized clinical trial seeks to directly test whether moral elevation is simply correlated with both (psychopathic trait scores and prosocial behavior on the game) or instead, is one of the mechanisms through which those with psychopathic traits exhibit less prosocial behavior.

## 2. Materials and methods

The Colorado Multiple Institutions Review Board (COMIRB #17–0182) approved the study protocol and the study protocol was registered with ClinicalTrials.gov (NCT03834467).

### 2.1 Recruitment, randomization and study procedures

**Fig 1:**
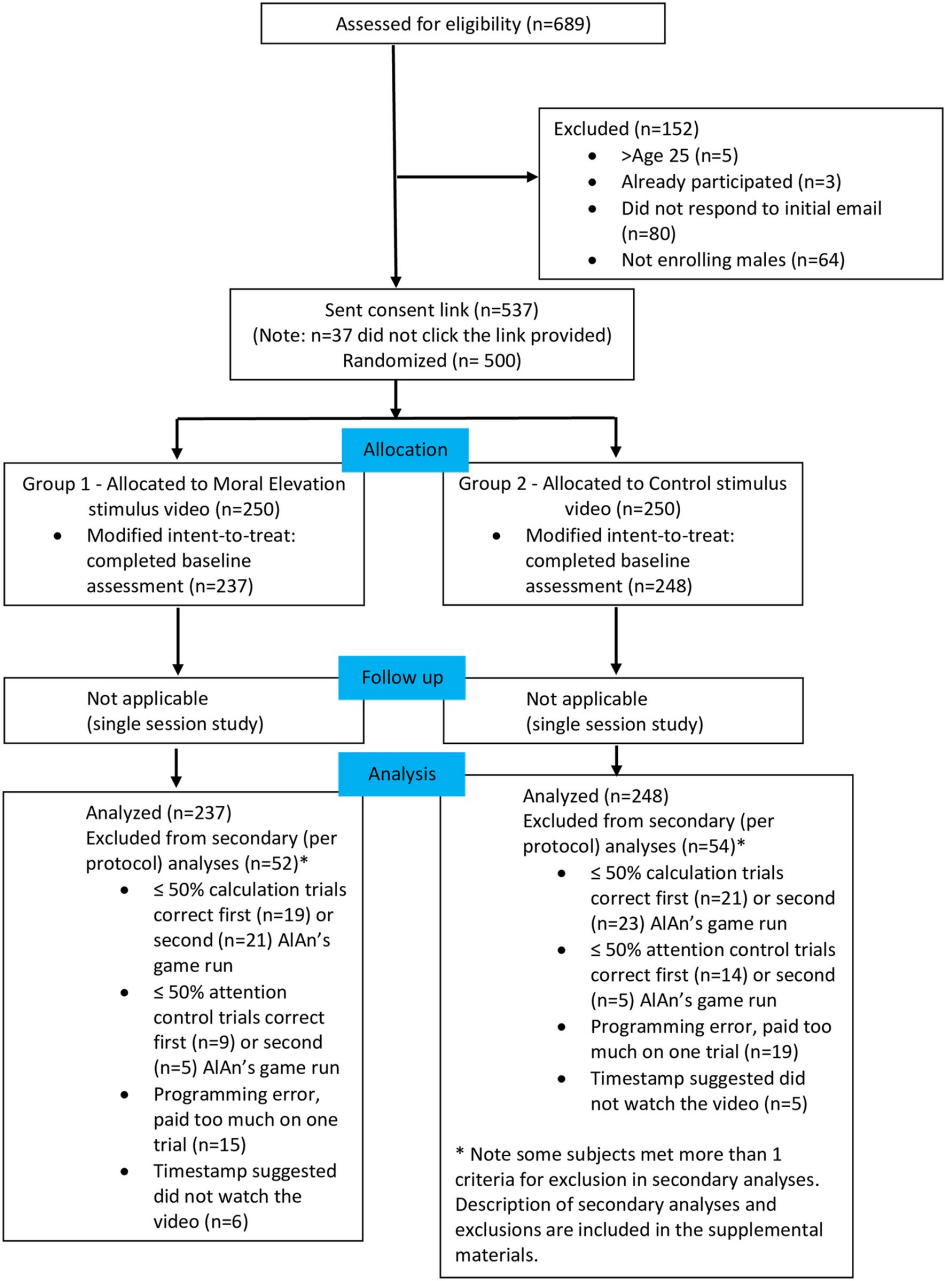
Consort flow diagram.

Subjects (n = 500) were recruited via advertising on Craigslist (some subjects contacted the study team via review of the ClinicalTrials.gov database), were assessed for meeting the study age requirement (18–25 years; note we sought to recruit a general population sample of young adults and therefore had minimal inclusion/exclusion criteria) and consented to study participation. The study statistician created a within-sex permuted blocks randomization scheme to assign subjects to Group (moral elevation vs. Control Stimulus video) that was implemented in REDCap [32], which allowed all (participants, investigators) except the study research assistant to remain blind to assignment. Subjects who consented to participation were provided with a link to complete the study protocol online via REDCap by the study research assistant. All subjects completed the Levenson Self-Report Psychopathy Scale [33], played the AlAn’s Short Game version 2 (v.2, Run 1; see below for a description), watched a video (Elevation stimulus, Group 1 vs. Control stimulus, Group 2, see below), played the AlAn’s Short Game v.2 again (Run 2), and completed a brief demographics questionnaire (see S1 Fig in S3 File), in that order. The modified intent to treat sample was determined by randomization plus completion of baseline assessment, because of the importance of adjusting for it, resulting in a sample of n = 485 (see Fig 1 and Table 1). Subjects were paid $20 plus earnings from playing the AlAn’s Short Game v.2.

**Table 1:** Comparisons of subject characteristics by study group.

	Group 1 (nature video; n = 248)	Group 2 (moral elevation video; n = 237)	Statistic; *p-value*
Age in years	21.68 (1.97)	21.70 (1.98)	*t*_483_ = -0.15; *p-value* = 0.88
Female gender	125 (50.4)	121 (51.1)	*χ*^2^_1_ = 0.02; *p-value* = 0.89
Race			
White	180 (72.6)	168 (70.9)	*Χ*^2^_6_ = 3.70; *p-value* = 0.72; *χ*^2^_2_ = 1.53; *p-value* = 0.47
Black	10 (4.0)	9 (3.8)	
Native American	2 (0.8)	5 (2.1)	
Asian	28 (11.3)	21 (8.9)	
Hispanic	15 (6.1)	15 (6.3)	
Other or more than one race	12 (4.8)	18 (7.6)	
Would rather not answer	1 (0.4)	1 (0.4)	
LSRPS Factor One	36.4 (8.41)	35.9 (8.26)	*t*_483_ = 0.67; *p-value* = 0.50
LSRPS Factor Two	21.7 (4.85)	22.1 (5.26)	*t*_483_ = -0.80; *p-value* = 0.42
LSRPS Total Score	58.1 (11.76)	58.0 (12.18)	*t*_479.89_ = 0.13; *p-value* = 0.90
RCVAS	85.5 (15.72)^c^	84.9 (16.03)^d^	*MW*; *p-value* = 0.63

Mean (standard deviation) or Count (%). For testing, race was collapsed into 3 categories: white, non-white, and other/no answer. MW = Mann-Whitney U test; LSRPS = Levenson Self Report Psychopathy Scale; RCVAS = Red Cross Visual Analog Score (0–100, higher scores indicate a more positive view of the Red Cross).

### 2.2 AlAn’s short game version 2

**Fig 2:**
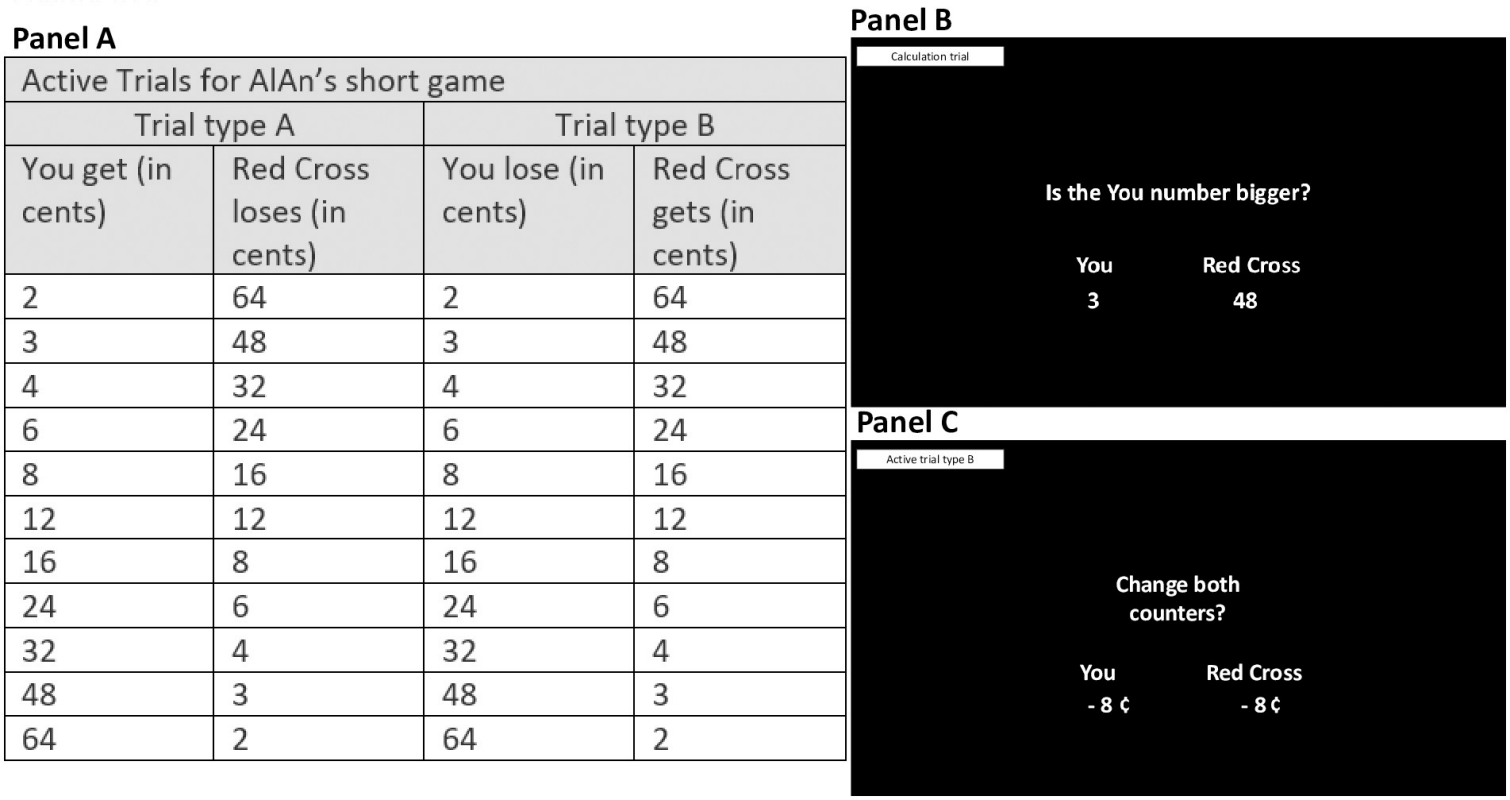
AlAn’s short game version 2 description. **Panel A** shows the active trial types presented while playing the game. They include trials where “You” gain money but the Red Cross donation is reduced (Active Trial type A). These trial types have been included in previous versions of the game. We also include trials where the participant (“You”) lose money but the Red Cross donation is increased (Active Trial type B, new to version 2) and Calculation Trials (**Panel B**) which ask, “Is the You number bigger?” and present a “You” and “Red Cross” number. **Panel C** shows an example of an Attention Control Trial.

The AlAn’s short game version 2 is programmed in REDCap. Subjects start with $2.50 and the Red Cross donation starts at $2.50. There are three trial types in the game. (1) Active Trials ask subjects if they want to “Change both counters?” and provide a “You” number and a “Red Cross” number. Participants are asked to accept or reject each trial. Some Active Trials (Active Trial Type A), where “You” will gain money but the Red Cross Donation will go down (see Fig 2, Panel A, left 2 columns), have been used in previous versions of the game [17]. And we added a new trial type, Active Trial Type B, where the subject will lose money but the Red Cross donation will increase (Fig 2, Panel A, right two columns). Each offer (each row of panel A columns 1–2 and 3–4) is made once while playing the game. For our measure of prosocial behavior, we calculated the number of Active Trial Type A rejected (i.e. rejecting these trials represents a prosocial act) and separately, calculated the number of Active Trial Type B accepted (i.e. accepting these trials represents a prosocial act). (2) Calculation Trials test whether subjects understood the relative values used in the game. They ask “Is the You number bigger?” These trials use the same paired values as presented in Panel A, except the “You” and Red Cross values are equal (i.e., 12,12). There are 10 Calculation Trials in the game (see Fig 2, Panel B for an example of this kind of trial). (3) Attention Control Trials. We present subjects with a trial where both they and the Red Cross will lose money (-8/-8) and a trial where they both will gain money (+8/+8); both are presented twice for 4 attention control trials in each run). In this instance, where we anticipate that the Red Cross is viewed favorably, we do not expect subjects to accept the -8/-8 trials or reject the +8/+8 trials. Calculation and Attention Control Trials help us to identify subjects with potential problematic data (e.g., indicating the subject was not attending to trial information) and secondary per-protocol analyses exclude such subjects (see Supplemental Materials). Within the Alan’s game prior to beginning the study, the trial order of presentation was randomized and all subjects received the same trial order. Subjects played the game before (Run 1) and after (Run 2) watching the assigned video. These additional changes were made from our prior studies: we utilized a different video describing the Red Cross (https://www.youtube.com/watch?v=XFQDY8mMrUs), trial background was changed from blue to black, and an updated instructional video was created (https://www.youtube.com/watch?v=R1k48apJzBw&feature=youtu.be; this game instructional video was shown to subjects prior to playing the game). All subjects also completed a Red Cross visual analog scale (scores 0–100 with higher scores indicating a more favorable view of the charity) following our prior procedures [16, 17].

### 2.3 Elevation stimulus and control videos

Subjects were randomized to either view a moral elevation stimulus video (Group 1; available here (https://www.youtube.com/watch?v=_N2zhu5RH34&t=24s or a control nature video of similar length (Group 2; available here https://www.youtube.com/watch?v=9Qe1uL9Tzg8). The moral elevation stimulus video depicts a man risking his life to save another in the New York subway. This video stimulus has been used in prior moral elevation studies [16, 34] and has been shown to elicit significant increases in self-reported moral elevation when delivered using the methods employed here [17]. The nature video shows series of images of the ocean, green spaces and forests. We did not measure self-reported moral elevation and instead were interested in those assigned to the moral elevation stimulus vs. nature video as our exposure of interest. We made this decision because we have shown that the moral elevation video consistently elicits a self-reported moral elevation response [16, 17] and we were concerned that questionnaires used to evaluate moral elevation response contain questions that may prime more prosocial behavior (e.g., “The person in the story has shown me how to be a better person” and “I need to do more to help other people”, etc.)

### 2.4 Levenson Self Report Psychopathy Scale (LSRPS)

Prior to watching the assigned video, subjects completed the LSRPS, a self-report 26-item questionnaire, which provides a two-factor measure of psychopathic traits. We examined Factor 1 score (callousness; derived from 16 questions, range of scores 16–64), Factor 2 score (impulsive aggression; derived from 10 questions, range of scores 10–40) and Total score (sum of Factor 1 and 2 scores). This self-report measure has been previously validated in general [33] and in incarcerated [35] populations and has shown convergent and discriminative validity with other measures of psychopathic traits [36, 37]. Within our sample after appropriate reverse scoring, Cronbach’s alpha for the full 26 item scale was 0.87, for Factor 1 (16 items) was 0.85 and for Factor 2 (10 items) was 0.71.

### 2.5 Data analyses and study hypotheses

Using estimates from our prior work (standard deviation = 6.3) [17] we calculated that 200 subjects per Group would provide >80% power to detect a difference between Groups of at least 1.47 prosocial decisions as significant assuming an α = 0.05. Given concerns from our prior work about subjects not following all study procedures online (e.g., not watching the moral elevation stimulus completely [17], we recruited 250 subjects per group to allow for potential need to drop noncompliant subjects from some analyses. Although we did not power our study for the LSRPS correlations with game behavior *a priori*, with an alpha of 0.05 and a sample of 485, we had greater than 80% power to detect modestly sized correlations (r = 0.13).

Distributions of outcomes were assessed for approximate normality. Demographic characteristics were compared between Groups using independent t-tests and chi-square tests or their nonparametric equivalents as appropriate. Despite some directional hypotheses, all analyses specified significance level alpha = 0.05 two-tailed. Race was collapsed into 3 categories for inclusion in models as a covariate: white, non-white, and other/multiple/no answer. Sex was denoted as female or not female in models.

#### Examining performance of new trials where the player will lose money and the charity donation will increase

Using data from Run 1 only (prior to moral elevation stimulus vs. control exposure), we tested internal consistency (Kuder-Richardson 20) within and across Active Trial Type and examined the relationship between Active Trial Type A and Active Trial Type B using Pearson correlations initially. Then, a multiple regression of Active Trial Type B on Active Trial Type A for Run 1 estimated their association while adjusting for age, sex, race and Red Cross visual analog scale score.

#### Testing for a negative association between psychopathy trait scores and game behavior in Run 1

We first graphed trial acceptance rates by trial characteristics (i.e., the relative amounts to “You” and the Red Cross) and then examined acceptance rates for those with higher psychopathic trait scores (top 20%; note: this does not represent a clinical cut off but instead was an avenue through which to visualize behavioral patterns in the top quintile). We examined the relationships among prosocial behavior outcomes and psychopathic trait scores with Pearson correlations and then a multiple regression of prosocial behavior on psychopathic trait score, adjusting for age, race, sex and Red Cross visual analog scale score as well as the sex*psychopathic trait interaction if significant. We focused on LSRPS Factor 1 scores as our primary variable of interest in these analyses, in line with our prior work [17, 18].

#### Testing the effects of moral elevation on game behavior

We compared Groups over Run using a mixed model analysis of covariance (ANCOVA) adjusting for age, race, and Red Cross visual analog scale score with fixed effects of Group (moral elevation vs. nature video), Run (1 vs. 2), sex (female vs. not female; as well as potential interactions with other fixed effects) and Group by Run interaction and random effect of subject. The two repeated measures on subjects were assumed to have an unstructured covariance structure estimating separate variance for each run and covariance between them thereby circumventing the more restrictive compound symmetric covariance assumption required in repeated measures ANCOVA. Beginning with the 3-way interaction between Group, Run and sex, non-significant higher order interactions (*p*>0.05) were sequentially removed and the model rerun. The model including two-way interaction (i.e., Group*Run), as the effect of interest, and all lower order terms was the most parsimonious entertained.

#### Testing whether type of stimulus (moral elevation vs. Control) moderates the relationship between psychopathic traits and prosocial behavior

We hypothesized that the relationship between psychopathic trait score and prosocial behavior would be moderated by the type of video viewed in between Runs. We evaluated the influence of moral elevation video condition exposure in comparison to control video condition, using a mixed model ANCOVA adjusting for age, race, and Red Cross visual analog scale score with fixed effects of Group (moral elevation vs. nature video), Psychopathic trait score (Factor 1), Run (1 vs. 2) and sex, and all their interactions, and random effect of subject with covariance structure as before. Beginning with the 4-way interaction between Group, Psychopathic trait score, Run and sex, non-significant interactions were sequentially removed and the model rerun. The model including three-way interaction (i.e., Group*Psychopathic trait score*Run), as the effect of interest, and all lower order terms was the most parsimonious entertained.

## 3. Results

### Sample characteristics

Table 1 shows that subjects randomized to Group 1 (control nature video) and Group 2 (moral elevation video) were similar in terms of age, sex, race, and psychopathic trait scores.

### Examining performance of new trials where player loses money and the charity donation increases

We calculated internal consistency (Kuder-Richardson 20) within Run 1 for Active Trial Type A (0.83), for Active Trial Type B (0.81), and with appropriate reverse scoring of all Active Trial Type B, across Trial Types A and B (0.89). Behavior on the prior version game trials (the number of rejected Active Trial Type A) correlated strongly with newly introduced game trials (number of accepted Active Trial Type B; *r* = 0.71; *p-value*<0.001; n = 485; see S1 Table in S2 File; also see S2 Table in S2 File for within-sex correlations). After adjusting for age, sex, race and Red Cross visual analog scale score, multiple regression of number of accepted Active Trial Type B on number of Active Trial Type A rejected for Run 1 had an estimated slope of 0.69 (standard error of 0.032; *t*_484_ = 21.77; *p*<0.0001; partial Eta-Square = 0.50; n = 485). For subsequent analyses we summed the number of Active Type A Trials rejected and Active Type B trials accepted as our composite measure of prosocial behavior.

#### Testing for a negative association between Psychopathy trait scores and game behavior

**Fig 3:**
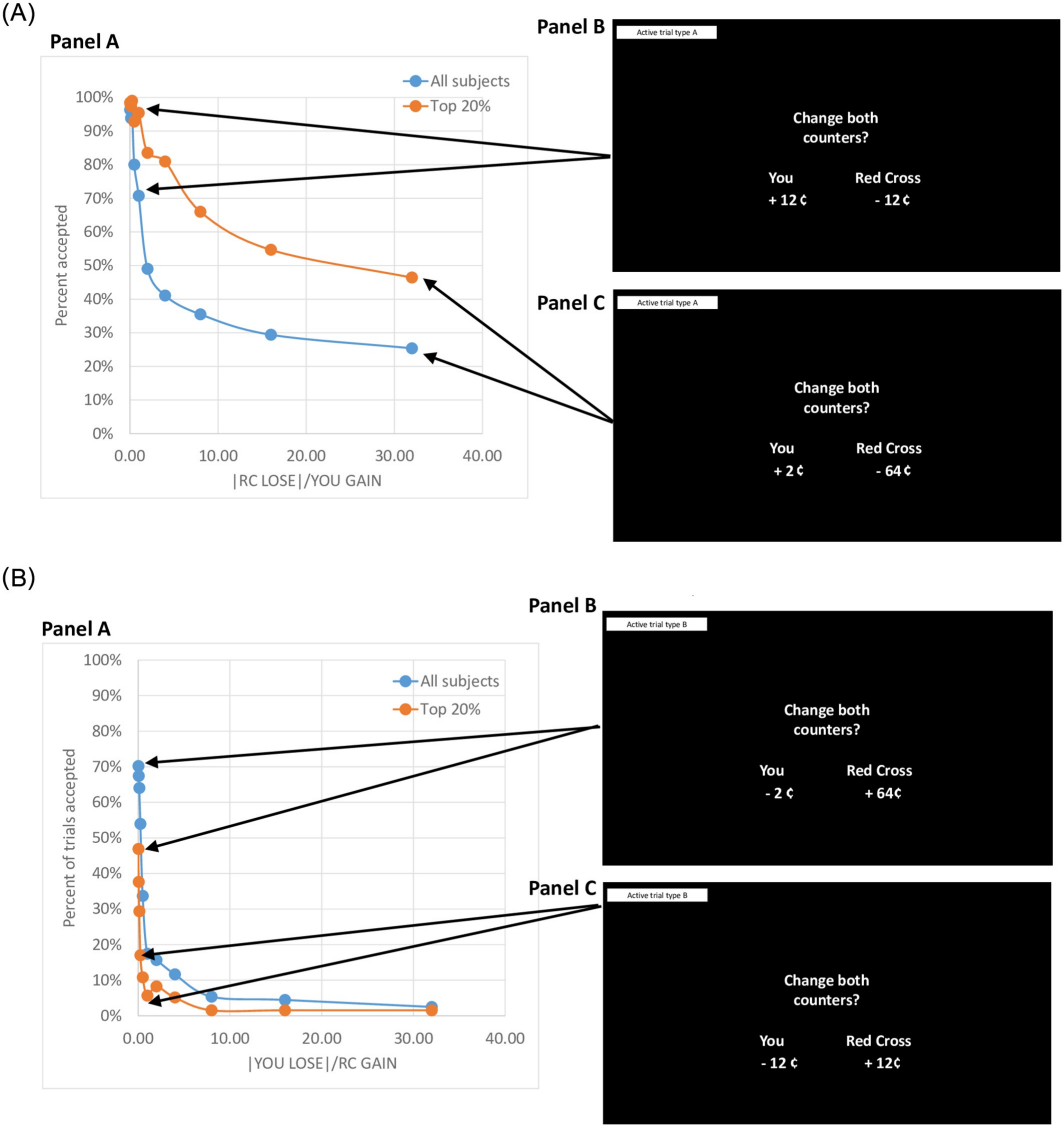
**A. Panel A**: Graphing Active Trials Type A (Red Cross loss and “You” gain trials)–Percent of trials accepted (Y-Axis) by Ratio of absolute value of Red Cross loss divided by “You” gain amount (X-Axis). Blue line indicates all subjects (n = 485 depending on the trial) and **orange line represents those in the top 20% for Levenson Self-Report Psychopathy Scale Factor 1 scores** (n = 97, with scores ≥45). **Panel B** shows Active Trial Type A with a ratio = 1; arrows show that these trials were accepted 71% and 95% of the time by the full sample and high Psychopathic traits group, respectively. **Panel C** shows Active Trial Type A with a ratio of 32; arrows show that these trials were accepted 25% and 46% of the time by the full sample and high Levenson Factor 1 score group, respectively. Note data from Runs 1 and 2 were included in these analyses. **B. Panel A**: Graphing Active Trials Type B (“You” lose and Red Cross gain trials)–Percent of trials accepted (Y-Axis) by Ratio of absolute value of “You” loss divided by Red Cross gain amount. Blue line indicates all subjects (n = 485) and **orange line represents those in the top 20% for Levenson Self Report Psychopathic Trait Factor 1 scores** (n = 97, with scores ≥45). **Panel B** shows Active Trial Type B with a ratio of 0.03; arrows show that these trials were accepted 70% and 47% of the time by the full sample and high Levenson Factor 1 group, respectively. **Panel C** shows Active Trial Type B with a ratio = 1; arrows show that these trials were accepted 17% and 6% of the time by the full sample and by the high Levenson Factor 1 score subset, respectively. Note data from Runs 1 and 2 were included in these analyses. Note: if self-benefit/loss and Red Cross loss/benefit were estimated by subjects to be of equal value, at the ratio = 1 (see X-Axis) we would expect trial acceptance rates of 50%. All comparisons at ratio = 1 (Red Cross loses 12¢ and “You” get 12¢ -- > Run 1 and Run 2, 18% and 17% acceptance rates, respectively; “You” lose 12¢ and Red Cross gets 12¢ -- > Run 1 and Run 2 accepted 72% and 69%, respectively) significantly differed from the expected 50% acceptance rate via binomial tests (all *p-values*<0.0001).

To describe game behavior for **Active Trial Type A** we graphed the percent of trials accepted (Y-Axis) by the trial information (absolute value of Red Cross loss divided by the amount the subject would receive) across Runs 1 and 2. As shown in Fig 3 panel A (blue line represents all subjects) as the ratio increases on the X-axis (more other harm, less reward to self), acceptance rates on the Y-axis decline. Behavior for subjects in the top 20% of the sample in terms of Factor 1 Psychopathic trait scores is shown by the orange line in Fig 3 panel A; acceptance rates for this group are consistently higher (e.g., more likely to accept offers where they will benefit but at a cost to the charity). We similarly graphed behavior for **Active Trial Type B** (see Fig 3 panel B) with the Y Axis showing acceptance rates and the X Axis the ratio of absolute value of “your” loss divided by the gain to the charity. Again, as the ratio increases (i.e., greater loss to self, less benefit to the charity) acceptance rates decline. Those scoring in the top 20% of the sample in terms of Factor 1 Psychopathic Trait scores generally had lower acceptance rates (i.e., less likely to accept trials where they would give up money to benefit the charity). Prosocial behavior from Run 1 negatively correlated with Factor 1 scores from the Levenson Self Report Psychopathy Scale (*r* = -0.52; *p-value*<0.001; see S1 Table in S2 File). In the multiple regression of prosocial behavior, adjusting for age, race, sex and Red Cross visual analog scale score, the Factor 1 psychopathic trait score*sex interaction was significant (*F*_1,477_ = 9.15; *p* = 0.003; partial Eta square = 0.02) such that the negative association in females (adjusted slope = -0.36) was greater than that in males (adjusted slope = -0.20), and the Factor 1 psychopathic trait score main effect t was also significant *(F*_1,477_ = 147.25; *p*<0.0001; partial Eta squared = 0.24).

#### Testing the effects of moral elevation on game behavior

**Fig 4:**
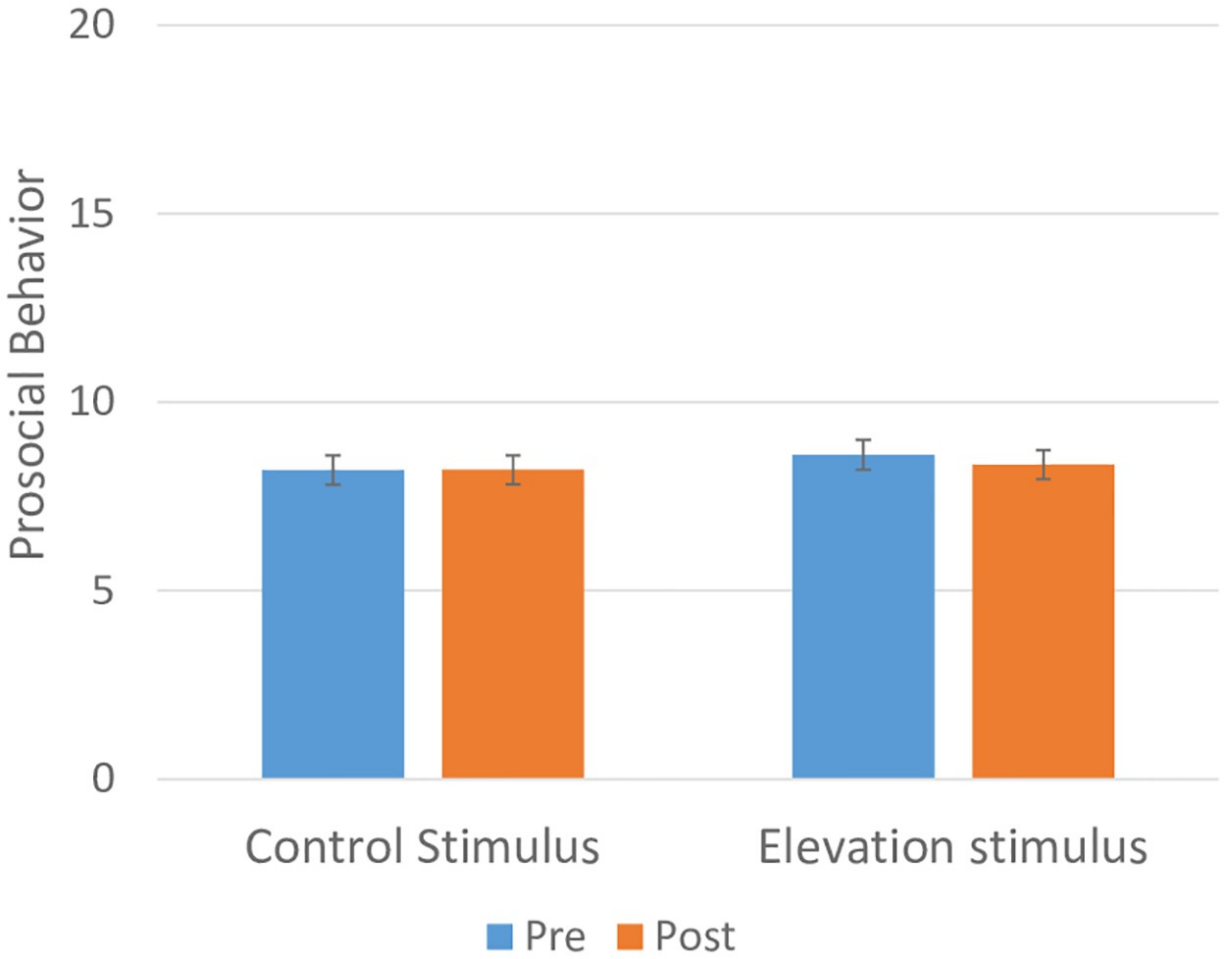
Testing for pre-stimulus to post-stimulus change in prosocial behavior among those receiving the moral elevation video stimulus (n = 237) vs. those receiving the Control (nature) video stimulus (n = 248) after adjusting for participant characteristics using a mixed model ANCOVA). A Group by Run interaction was non-significant (*F*_1,478_ = 2.40; *p* = 0.12). Adjusted estimates of prosocial behavior are shown with standard error.

As shown in Fig 4, both Groups (control and moral elevation video) showed relatively stable levels of prosocial behavior across runs 1 and 2. In a mixed model ANCOVA all interactions with sex were non-significant and removed sequentially as described. The Group by Run interaction was non-significant (*F*_1, 478_ = 2.40; *p* = 0.122) indicating that there was no significant difference in change in prosocial behavior between Groups after adjusting for participant characteristics; main effects of Group (*F*_1, 478_ = 0.42; *p* = 0.52) and Run (*F*_1,478_ = 2.25; *p* = 0.13) were also non-significant. The game showed good immediate test-retest reliability (S2 Fig in S1 File).

#### Testing whether moral elevation moderates the relationship between psychopathic traits and prosocial behavior

Analyses examining whether type of stimulus exposure moderated the negative association between psychopathic traits and prosocial behavior were non-significant, indicating that moral elevation exposure did not explain this relationship. In a mixed model ANCOVA evaluating effects of psychopathy, group, Run and sex after adjusting for participant characteristics, all higher order interactions were non-significant and sequentially removed, as described above. The psychopathic trait score by Group by Run interaction was non-significant (*F*_1,474_ = 0.10; *p* = 0.76), indicating that exposure to moral elevation stimulus, compared to control, did not moderate the negative relationship between psychopathic trait score and prosocial behavior. Of the main effects only Factor 1 Psychopathic trait score (*F*_1,474_ = 149.26; *p*<0.0001) was significantly associated with prosocial behavior (Run *F*_1,474_ = 0.67; *p* = 0.41; Group *F*_1,474_ = 0.00; *p* = 0.99).

Sensitivity analyses removing some participants are reviewed in the Supplemental Materials; results were similar to those from our primary modified intent to treat sample.

## 4. Discussion

This revised version of the AlAn’s game provides useful information about self-other considerations and allows mapping of acceptance rates by self-other valuations (see Fig 3). Our study provides new information by including trials where the subject will lose money and the charity will gain money. Accepting Active Trial Type A (i.e., benefitting self and costing another) might be framed as an antisocial behavior, while accepting Active Trial Type B (i.e., trials that cost self and benefit another) aligns with prior definitions of altruism. However, these two trial types showed good internal consistency across items and were strongly correlated with one another, suggesting that these trials are considered similarly. As such, our results are in line with antisocial and altruistic behavioral tendencies (as defined here) potentially being considered on a spectrum (e.g., an individual who tends to behave in ways that cost others to benefit self appears to also be less likely to engage in behaviors that help other at a cost to oneself).

Both Active Trial Type A and new Active trial Type B suggest that on average there is a bias toward over-valuing oneself over the charity. For example, if self-benefit/loss and Red Cross loss/benefit were estimated by subjects to be of equal value, at the ratio = 1 (see Fig 3, panels A and B, ratio on the X-Axis) we would expect trial acceptance rates to average 50%. Here there is a preference both for Trial Type A (higher acceptance rates) and the new Trial Type B (lower than 50%)–suggesting a bias toward more heavily valuing self-relative to the charity. In addition, behavior on Runs 1 and 2 of the AlAn’s game were highly correlated (see S2 Fig in S3 File) supporting a high immediate test-retest reliability and suggesting (assuming that behavior on the game is modifiable) that this game provides a useful platform for testing change in prosocial behavior (e.g., with high immediate test-retest reliability small changes can be detected with smaller samples). However, future studies with longer durations between test to re-test would help to better delineate the stability of prosocial behavior using this paradigm. Finally, prosocial behavior on the game was negatively associated with psychopathic trait scores (see S1 Table in S2 File)–those with high psychopathic traits scores exhibit less prosocial behavior (see Fig 3)—supporting the validity of this measure of prosocial behavior at least in relation to this phenotype.

Unfortunately, we did not find that moral elevation stimulus exposure led to changes in prosocial behavior. Several prior studies support an effect of moral elevation on prosocial acts [6, 7, 27, 29, 31], though there are some exceptions e.g., for volunteering and the Ultimatum Game [6], and many of these prior studies suggest large effects of moral elevation on prosocial behavior (e.g., 69% vs. 40% volunteering to participate in an unpaid study; [31]; donating about 60% more [7]). It is therefore important to consider aspects of our design which may have contributed to this null result including: the paradigm utilized here measures one type of prosocial behavior and the effects of moral elevation seen in prior studies may be unrelated to such self-other considerations as framed by this paradigm; subjects may not have experienced a moral elevation response (Note: we did not measure self-reported moral elevation response based on prior results that the video successfully elicits self-reported moral elevation [16, 17] and out of concern that querying moral elevation response might in itself prime subjects to act more prosocially). By utilizing online data collection, we cannot be assured that subjects viewed the video, though time-stamps help to reduce this concern. It is also possible that the Red Cross video, which we utilize to introduce the charity to subjects, might have elicited a moral elevation response itself. This may have dampened effects from the moral elevation stimulus. In addition, the control video may have elicited “awe” which can impact prosocial behavior [38] (though this explanation appears less likely given the lack of change in prosocial behavior in the control group; i.e., see S2 Fig in S3 File, panel A). Future studies might explore these potential explanations. Finally, it is important to note that this study utilized a general population sample, instead of recruiting individuals at the extreme end of the distribution for this trait, and we examined psychopathic trait scores as a continuous measure.

Enhancement of prosocial behavior and understanding the mechanisms which lead to such enhancements is significant. Prosocial behavior is negatively related to both externalizing and internalizing disorders [39–45] and is positively related to a broad set of positive outcomes: happiness [46], self-esteem [47], good academic performance [48], better peer relationships [49] and is protective against future loneliness [50]. Other potential motivators toward prosocial behavior should also be explored. For example, some have suggested that loving-kindness/mindfulness meditation might impact prosocial behavior, not through the experience of positive emotions, but through priming toward compassion and empathy [51]. Others have suggested that promoting mentalization and perspective taking in children can enhance context specific prosocial behaviors [52]. Still others have suggested that prosocial behavior requires top-down control (shutting off prepotent tendency to act in a self-oriented manner). Some data has suggested taxing cognitive control causes decrements in prosocial behavior [53]. Such other targets should be systematically explored to better understand whether, under randomized, controlled and blinded conditions, certain effects can be shown to consistently influence within-subject change in prosocial decision making. Such work has broad implications, including potential avenues for investigation of underlying mechanisms which contribute to clinical phenotypes (e.g., Psychopathy).

## 5. Conclusions

Behavior on the AlAn’s game v.2 is related to psychopathic trait scores. The game also shows high correlations between runs. Utilizing a randomized, controlled design, we were unable to demonstrate that exposure to a moral elevation stimulus video changes performance on a previously validated game of prosocial behavior (self-other considerations). Further study of the effects of moral elevation on prosocial behavior are merited, along with studies of other potential variables increasing prosocial behavior.

## Supporting information

S1 File (DOCX)

S2 File (DOCX)

S3 File (PPTX)

S4 File (DOC)
