# Supplementary material for: A revised prosocial behavior game: Testing associations with psychopathic traits and the effects of moral elevation using a randomized clinical trial
Source: PLoS One. 2023 Apr 19;18(4):e0283279. doi: 10.1371/journal.pone.0283279 (PMC10115303; doi:10.1371/journal.pone.0283279)
Supplement: S1 File — (DOCX) [file pone.0283279.s001.docx]

**SUPPLEMENTAL INFORMATION**

**Further description of the AlAn’s game and design rational.**

The AlAn’s (Altruism-Antisocial) game was originally tested in adolescent populations and the term “game” was utilized, instead of “test” or “paradigm,” as we considered this term potentially more engaging for adolescent participants. The AlAn’s game asks participants to make decisions which require weighing self vs. other valuations and was constructed to examine a broad range of relative values (self vs. other). Prior work supports that it is the relative, rather than absolute values, that drive such self:other decision making (1, 2). For example, Zaki & Mitchell (2011) suggested that individuals not only estimate the value of a benefit to self but use similar mechanisms to also estimate value that arises from behaviors, which enforce equity and fairness (i.e., a “person invariant” system (3)). Under this model, individuals will act prosocially when the estimated value from fairness and equity enforcement exceeds the estimated value from self-benefit (1). Individuals appear to engage in such valuations even when limited information is available regarding the other (1). But when additional information is available, such estimates appear to include determinations regarding the other to be helped/harmed (e.g., in group vs. out group; justice enforcement; estimates of deservingness (4-8)). For example, in the dictator game, players are often willing to give up a monetary gain to enforce a cost to someone who is viewed as a “bad actor,” a tendency previously termed Costly Punishment (6, 9). And similarly, individuals appear to often be willing to give up a benefit to help or protect an individual who is viewed as a “good actor,” which has been termed Costly Helping (10).

We utilized a charity that was likely to be viewed positively and had subjects view a video about the charity to ensure each subject had similar information about the “other” in the game. We measured (Red Cross Visual Analog Scale) participant perceptions about the Red Cross prior to playing the game and include this as a covariate in analyses. Based on the model described above, we visualize decision making using relative valuations (e.g., see x-axes in Figure 3 graphs) and utilize a range of relative values that provide a comprehensive description of the decision making curve (e.g., adding trials with ratios further to the right on the x-axis in the graphs in Figure 3, would likely add little information as the curve has essential reached an asymptote in panel A, and a floor restriction in panel B. Though we considered utilizing large dollar amounts with a single trial being randomly selected to be “real”, in the AlAn’s game all decisions are real but of small dollar amounts; we made this choice because of (a) the importance of relative, rather than absolute, valuations (see paragraph above) and (b) given our interest in examining antisocial phenotypes and the clear association between antisocial phenotypes (and personality traits) and differential assessment of risk/probabilities (11, 12). The game was constructed to be a single player game and to not allow for reciprocity (e.g., I am prosocial to you with the hope that you will be prosocial in return). Finally, players are explicitly told that how they played the game will not be shared with others. Thus, in the game prosocial decisions are made without the expectation of reciprocal altruism or clear effects on reputation in their day-to-day life.

**Additional analyses examining the effects of Trial Type on the relationship between Levenson Factor 1 score and prosocial behavior on the game.** We completed an additional analysis, based on helpful reviewer feedback, to test whether Trial Type (A vs. B) differentially impacted the relationship between Psychopathic traits and prosocial behavior. We ran a multiple regression of prosocial behavior during run 1 on psychopathic trait score, adjusting for age, race, sex, Red Cross visual analog scale score and Trial Type as well as the Trial Type*psychopathic trait interaction. This interaction was non-significant (F_1_=2.45; p=0.1179) but Levenson Factor 1 score (F_1_=162.94; p<0.0001) and Trial Type (F_1_=4.65; p=0.0315) were significant. Least square means for Trial Type A (4.08 prosocial choices) and Trial Type B (3.83 prosocial choices) suggested a slightly lower number of prosocial behaviors on Trial Type B.

**Sensitivity analyses re-conducting our main analyses while excluding subjects for various reasons**. Hypotheses were evaluated first in the modified-intent-to-treat sample (n=485; see main manuscript for those analyses). Analyses were then repeated removing individuals who had poor performance on Calculations and Attention Control trials (≤50% correct), those who did not completely watch the study videos (Elevation/Control stimulus or instructions based on time stamps placed within REDCap), and subjects who received a larger payment for a single Attention Control Trial (64 cents instead of 8 cents, due to a programming error found early in the study), resulting in a subsample of n=379.

Testing the effects of moral elevation on game behavior. We compared Groups over Run using a mixed model ANCOVA adjusting for age, race, and Red Cross visual analog scale score with fixed effects of Group (moral elevation vs. nature video), Run (1 vs. 2), sex (and its potential interactions with fixed effects) and Group by Run interaction and random effect of subject. The two repeated measures on subjects were assumed to have an unstructured covariance structure estimating separate variance for each run and covariance between them. Beginning with the 3-way interaction between Group, Run and sex, non-significant higher order interactions (p>0.05) were sequentially removed and the model rerun. The model including two-way interaction (i.e., Group*Run), as the effect of interest, and all lower order terms was the most parsimonious entertained.

All interactions with sex were non-significant and removed sequentially as described. The Group by Run interaction was non-significant (*F*_1, 372_ =0.38; *p*=0.535) indicating that there was no significant difference in change in prosocial behavior between Groups after adjusting for participant characteristics.

REFERENCES:

1. Zaki J, López G, Mitchell JP. Activity in ventromedial prefrontal cortex co-varies with revealed social preferences: evidence for person-invariant value. Social cognitive and affective neuroscience. 2014;9(4):464-9.

2. Sakai JT, Raymond KM, McWilliams SK, Mikulich-Gilbertson SK. Testing helping behavior and its relationship to antisocial personality and psychopathic traits. Psychiatry research. 2019;274:98-104.

3. Zaki J, Mitchell JP. Equitable decision making is associated with neural markers of intrinsic value. Proceedings of the National Academy of Sciences. 2011;108(49):19761-6.

4. Eckel CC, Grossman PJ. Altruism in anonymous dictator games. Games and economic behavior. 1996;16(2):181-91.

5. Fehr E, Gächter S. Altruistic punishment in humans. Nature. 2002;415(6868):137-40.

6. Knoch D, Gianotti LR, Baumgartner T, Fehr E. A neural marker of costly punishment behavior. Psychological science. 2010;21(3):337-42.

7. Sparks E, Schinkel MG, Moore C. Affiliation affects generosity in young children: The roles of minimal group membership and shared interests. Journal of Experimental Child Psychology. 2017;159:242-62.

8. Wedekind C, Milinski M. Cooperation through image scoring in humans. Science. 2000;288(5467):850-2.

9. Henrich J, McElreath R, Barr A, Ensminger J, Barrett C, Bolyanatz A, et al. Costly punishment across human societies. Science. 2006;312(5781):1767-70.

10. Sakai JT, Dalwani MS, Mikulich-Gilbertson SK, McWilliams SK, Raymond KM, Crowley TJ. A behavioral measure of costly helping: Replicating and extending the association with callous unemotional traits in male adolescents. PloS one. 2016;11(3):e0151678.

11. Crowley TJ, Raymond KM, Mikulich-Gilbertson SK, Thompson LL, Lejuez CW. A risk-taking “set” in a novel task among adolescents with serious conduct and substance problems. Journal of the American Academy of Child & Adolescent Psychiatry. 2006;45(2):175-83.

12. Lauriola M, Levin IP. Personality traits and risky decision-making in a controlled experimental task: An exploratory study. Personality and individual differences. 2001;31(2):215-26.
