# Supplementary material for: A revised prosocial behavior game: Testing associations with psychopathic traits and the effects of moral elevation using a randomized clinical trial
Source: PLoS One. 2023 Apr 19;18(4):e0283279. doi: 10.1371/journal.pone.0283279 (PMC10115303; doi:10.1371/journal.pone.0283279)
Supplement: S2 File — (DOCX) [file pone.0283279.s002.docx]

| SUPPLEMENTAL TABLE 1. Pearson Correlation Matrix of AlAn’s Game Trials and Levenson Self Report Psychopathy Scale Scores (all *p-values* <0.001; n=485) | | | | | | | | | | |
| --- | --- | --- | --- | --- | --- | --- | --- | --- | --- | --- |
|  | Prosocial Behavior Trial Type A^1^ (number rejected, Run 1) | Prosocial Behavior Trial Type B^2^ (number accepted, Run 1) | Prosocial Behavior Both Trial Types combined (Run 1) | Prosocial Behavior Trial Type A (number rejected, Run 2) | Prosocial Behavior Trial Type B (number accepted, Run 2) | Prosocial Behavior Both Trial Types combined (Run 2) | Prosocial Behavior Both Trial Types Combined (Runs 1 and 2) | Levenson Factor 1 Score | Levenson Factor 2 Score | Levenson Total Score |
| Prosocial Behavior Trial Type A (Run 1) | 1 | *r*=0.71 | *r*=0.93; | *r*=0.90 | *r*=0.72 | *r*=0.87 | *r*=0.92 | *r*= -0.50 | *r*= -0.30 | *r*= -0.47 |
| Prosocial Behavior Trial Type B (Run 1) |  | 1 | *r*=0.92 | *r*=0.68 | *r*=0.88 | *r*=0.84 | *r*=0.90 | *r*= -0.46 | *r*= -0.32 | *r*= -0.46 |
| Prosocial Behavior Both Trial Types combined (Run 1) |  |  | 1 | *r*=0.86 | *r*=0.86 | *r*=0.92 | *r*=0.98 | *r*= -0.52 | *r*= -0.33 | *r*= -0.50 |
| Prosocial Behavior Trial Type A (Run 2) |  |  |  | 1 | *r*=0.74 | *r*=0.94 | *r*=0.92 | *r*= -0.49 | *r*= -0.29 | *r*= -0.46 |
| Prosocial Behavior Trial Type B (Run 2) |  |  |  |  | 1 | *r*=0.93 | *r*=0.91 | *r*= -0.44 | *r*= -0.34 | *r*= -0.45 |
| Prosocial Behavior Both Trial Types combined (Run 2) |  |  |  |  |  | 1 | *r*=0.98 | *r*= -0.50 | *r*= -0.33 | *r*= -0.49 |
| Prosocial Behavior Both Trial Types Combined (Runs 1 and 2) |  |  |  |  |  |  | 1 | *r*= -0.52 | *r*= -0.34 | *r*= -0.51 |
| Levenson Factor One Score |  |  |  |  |  |  |  | 1 | *r*=0.57 | *r*=0.94 |
| Levenson Factor Two Score |  |  |  |  |  |  |  |  | 1 | *r*=0.82 |

^1^ Trial Type A = where the participant will gain money but the Red Cross donation will lose money. Prosocial behavior for these trials is defined as the number of trials rejected.

^2^ Trial Type B = where the participant will lose money but the Red Cross donation will gain money. Prosocial behavior for these trials is defined as the number of trial accepted.

| SUPPLEMENTAL TABLE 2. Pearson Correlation Matrix of AlAn’s Game Trials and Levenson Self Report Psychopathy Scale Scores within females (n=246) above, within and males (n=239); all *p-values*<0.001. | | | | | | | | | | |
| --- | --- | --- | --- | --- | --- | --- | --- | --- | --- | --- |
|  | Prosocial Behavior Trial Type A (number rejected, Run 1) | Prosocial Behavior Trial Type B (number accepted, Run 1) | Prosocial Behavior Both Trial Types combined (Run 1) | Prosocial Behavior Trial Type A (number rejected, Run 2) | Prosocial Behavior Trial Type B (number accepted, Run 2) | Prosocial Behavior Both Trial Types combined (Run 2) | Prosocial Behavior Both Trial Types Combined (Runs 1 and 2) | Levenson Factor 1 Score | Levenson Factor 2 Score | Levenson Total Score |
| Prosocial Behavior Trial Type A (Run 1) | 1 | *r_MALE_*=0.75  *r_FEMALE_*=0.68 | *r_MALE_*=0.94  *r_FEMALE_*=0.92 | *r_MALE_*=0.91  *r_FEMALE_*=0.89 | *r_MALE_*=0.72  *r_FEMALE_*=0.71 | *r_MALE_*=0.88  *r_FEMALE_*=0.87 | *r_MALE_*=0.92  *r_FEMALE_*=0.91 | *r_MALE_*= -0.31  *r_FEMALE_*= -0.66 | *r_MALE_*= -0.24  *r_FEMALE_*= -0.36 | *r_MALE_*= -0.32  *r_FEMALE_*= -0.61 |
| Prosocial Behavior Trial Type B (Run 1) |  | 1 | *r_MALE_*=0.93  *r_FEMALE_*=0.91 | *r_MALE_*=0.71  *r_FEMALE_*=0.66 | *r_MALE_*=0.89  *r_FEMALE_*=0.87 | *r_MALE_*=0.85  *r_FEMALE_*=0.82 | *r_MALE_*=0.91  *r_FEMALE_*=0.88 | *r_MALE_*= -0.36  *r_FEMALE_*= -0.54 | *r_MALE_*= -0.27  *r_FEMALE_*= -0.36 | *r_MALE_*= -0.37  *r_FEMALE_*= -0.53 |
| Prosocial Behavior Both Trial Types combined (Run 1) |  |  | 1 | *r_MALE_*=0.87  *r_FEMALE_*=0.85 | *r_MALE_*=0.86  *r_FEMALE_*=0.86 | *r_MALE_*=0.92  *r_FEMALE_*=0.92 | *r_MALE_*=0.98  *r_FMEALE_*=0.98 | *r_MALE_*= -0.36  *r_FMEALE_*= -0.65 | *r_MALE_*= -0.27  *r_FEMALE_*= -0.40 | *r_MALE_*= -0.37  *r_FEMALE_*= -0.63 |
| Prosocial Behavior Trial Type A (Run 2) |  |  |  | 1 | *r_MALE_*=0.74  *r_FEMALE_*=0.74 | *r_MALE_*=0.93  *r_FEMALE_*=0.94 | *r_MALE_*=0.92  *r_FEMALE_*=0.91 | *r_MALE_*= -0.32  *r_FEMALE_*= -0.64 | *r_MALE_*= -0.24  *r_FEMALE_*= -0.34 | *r_MALE_*= -0.32  *r_FEMALE_*= -0.59 |
| Prosocial Behavior Trial Type B (Run 2) |  |  |  |  | 1 | *r_MALE_*=0.93  *r_FEMALE_*=0.93 | *r_MALE_*=0.91  *r_FEMALE_*=0.91 | *r_MALE_*= -0.36  *r_FEMALE_*= -0.53 | *r_MALE_*= -0.29  *r_FEMALE_*= -0.39 | *r_MALE_*= -0.37  *r_FEMALE_*= -0.53 |
| Prosocial Behavior Both Trial Types combined (Run 2) |  |  |  |  |  | 1 | *r_MALE_*=0.98  *r_FEMALE_*=0.98 | *r_MALE_*= -0.36  *r_FEMALE_*= -0.63 | *r_MALE_*= -0.28  *r_FEMALE_*= -0.39 | *r_MALE_*= -0.37  *r_FMEALE_*= -0.60 |
| Prosocial Behavior Both Trial Types Combined (Runs 1 and 2) |  |  |  |  |  |  | 1 | *r_MALE_*= -0.37  *r_FEMALE_*= -0.65 | *r_MALE_*= -0.28  *r_FEMALE_*= -0.40 | *r_MALE_*= -0.38  *r_FEMALE_*= -0.63 |
| Levenson Factor One Score |  |  |  |  |  |  |  | 1 | *r_MALE_*=0.57  *r_FEMALE_*=0.57 | *r_MALE_*=0.93  *r_FEMALE_*=0.94 |
| Levenson Factor Two Score |  |  |  |  |  |  |  |  | 1 | *r_MALE_*=0.83  *r_FEMALE_*=0.81 |
| Levenson Total Score |  |  |  |  |  |  |  |  |  | 1 |

Trial Type A = where the participant will gain money but the Red Cross donation will lose money. Prosocial behavior for these trials is defined as the number of trials rejected.

Trial Type B = where the participant will lose money but the Red Cross donation will gain money. Prosocial behavior for these trials is defined as the number of trial accepted.
