# Supplementary material for: A revised prosocial behavior game: Testing associations with psychopathic traits and the effects of moral elevation using a randomized clinical trial
Source: PLoS One. 2023 Apr 19;18(4):e0283279. doi: 10.1371/journal.pone.0283279 (PMC10115303; doi:10.1371/journal.pone.0283279)
Supplement: S3 File — (PPTX) [file pone.0283279.s003.pptx]

## Slide 1
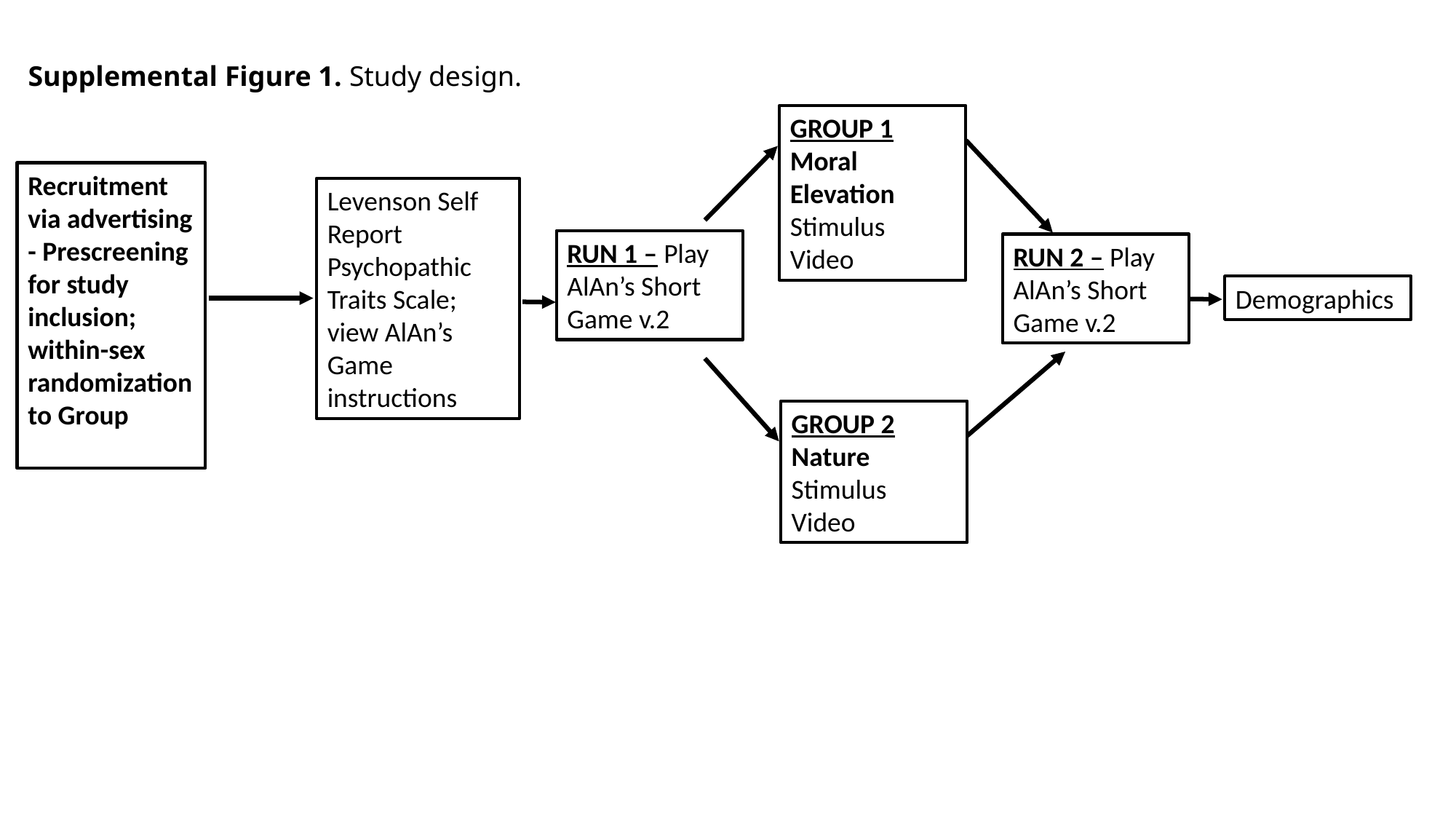

# Supplemental Figure 1. Study design.
GROUP 1
Moral Elevation Stimulus Video
Recruitment via advertising - Prescreening for study inclusion; within-sex randomization to Group
Levenson Self Report Psychopathic Traits Scale; view AlAn’s Game instructions
RUN 1 – Play AlAn’s Short Game v.2
RUN 2 – Play AlAn’s Short Game v.2
Demographics
GROUP 2
Nature Stimulus Video

## Slide 2
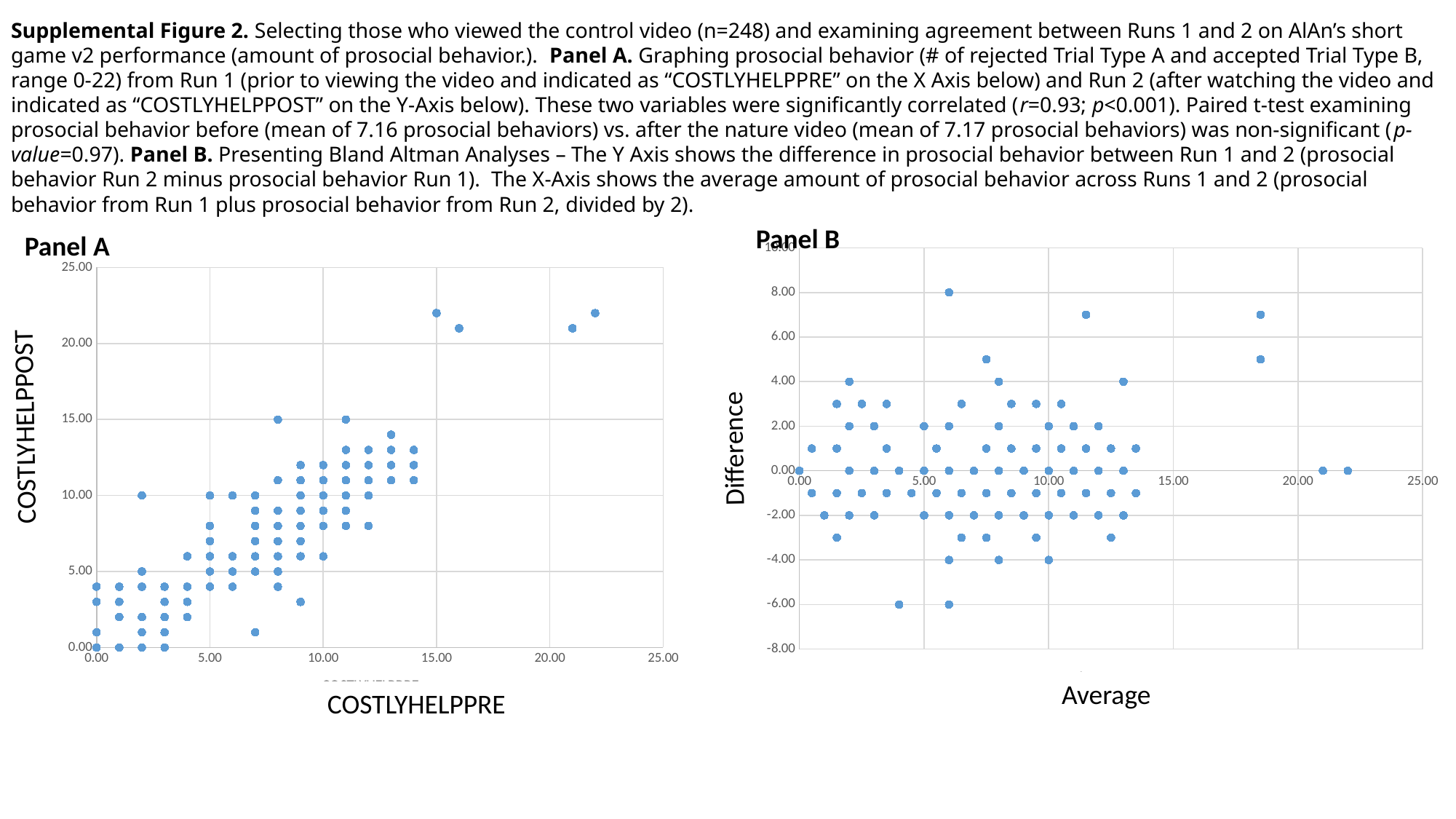

Supplemental Figure 2. Selecting those who viewed the control video (n=248) and examining agreement between Runs 1 and 2 on AlAn’s short game v2 performance (amount of prosocial behavior.). Panel A. Graphing prosocial behavior (# of rejected Trial Type A and accepted Trial Type B, range 0-22) from Run 1 (prior to viewing the video and indicated as “COSTLYHELPPRE” on the X Axis below) and Run 2 (after watching the video and indicated as “COSTLYHELPPOST” on the Y-Axis below). These two variables were significantly correlated (r=0.93; p<0.001). Paired t-test examining prosocial behavior before (mean of 7.16 prosocial behaviors) vs. after the nature video (mean of 7.17 prosocial behaviors) was non-significant (p-value=0.97). Panel B. Presenting Bland Altman Analyses – The Y Axis shows the difference in prosocial behavior between Run 1 and 2 (prosocial behavior Run 2 minus prosocial behavior Run 1). The X-Axis shows the average amount of prosocial behavior across Runs 1 and 2 (prosocial behavior from Run 1 plus prosocial behavior from Run 2, divided by 2).
Panel B
Panel A
### Chart:
| Category | |
|---|---|
### Chart:
| Category | |
|---|---|COSTLYHELPPOST
Difference
Average
COSTLYHELPPRE
