# Supplementary material for: A revised prosocial behavior game: Testing associations with psychopathic traits and the effects of moral elevation using a randomized clinical trial
Source: PLoS One. 2023 Apr 19;18(4):e0283279. doi: 10.1371/journal.pone.0283279 (PMC10115303; doi:10.1371/journal.pone.0283279)
Supplement: S4 File — (DOC) [file pone.0283279.s004.doc]

| w | *COMIRB Protocol* |  |
| --- | --- | --- |
| COLORADO MULTIPLE INSTITUTIONAL REVIEW BOARDCAMPUS BOX F-490 TELEPHONE: 303-724-1055 Fax: 303-724-0990 | |  |

**Protocol #: 17-0182**

**Project Title: Testing a shortened version of the AlAn’s Game, which can be delivered through REDCap, in a sample of emerging adults**

**Principal Investigator: Joseph T. Sakai, M.D.**

**Version Date: 11/20/2018**

**I**. **Hypotheses and Specific Aims**:

**Aim 1:** Examine the similarities and differences in behaviors on the AlAn’s Short Game v.2 seen with the two Active Trial (AT) types (where Red Cross donation will be reduced and you will gain money (AT1) vs. where the Red Cross donation will increase and you will lose money (AT2).

**Hypothesis 1:** Number of accepted trials of AT1 and AT2 from session 1 will significantly correlate.

**Aim 2:** Test the effects of Elevation vs. Nature video on change in AlAn’s Short Game v.2 behavior between sessions 1 and 2.

**Hypothesis 2:** Subjects who view the Elevation stimulus video (study Arm 1) will show increases in prosocial behavior while those watching the Nature video will not.

**Aim 3:** Test the association between psychopathic traits and Costly Helping and determine whether the influence of Elevation on Costly Helping dissipates with increasing levels of psychopathic traits.

**Hypothesis 3:** Psychopathic traits, as measured by the Levenson Self Report Psychopathy Scale, will significantly and negatively correlate with the amount of prosocial behavior in session 1.

**Hypothesis 4:** Using subjects from study Arm 1, change in AlAn’s Short game v.2 behavior (session 2 minus session 1) will be negatively correlated with psychopathic trait scores.

**II**. **Background and Significance**:

We have previously developed and tested the AlAn’s game (under COMIRB Protocol #07-0213, PI: Sakai), which asks subjects to repeatedly decide whether to accept or reject offers where they will benefit but a charity donation will be reduced. We have associated behavior on this simple game with measures of externalizing behavior problems and game behavior discriminates typically developing adolescents from those who meet the DSM-5’s “with limited prosocial emotions” (LPE) specifier. Those with LPE take more money for themselves and leave less money in the charity donation (Sakai et al., 2012; 2016). We have also utilized this game in the MRI to examine brain activation patterns, while subjects make decisions requiring weighing self-benefit and other-harm (COMIRB Protocol #12-0117, PI: Sakai / Sakai et al., under review).

While this early work is encouraging, the game had some limitations. These include: (1) the game takes about 30 minutes to play, (2) the game is run utilizing E-Prime software, which requires a license, and (3) the game has to be played in-person after set-up on a laptop by a research assistant. Thus, our studies to date have used relatively modest sample sizes. To address these concerns, we developed a shortened version of the game, now in its second revision which we call AlAn’s Short Game, version 2 (v.2), which can be played via the REDCap program and is described in detail below. Here we seek to test whether the second version of the game is related to phenotypes of interest (hypotheses 1-4 listed above).

In our prior work, we demonstrated that behavior on the AlAn’s game is related to Moral Elevation response. Moral Elevation is a positive response to another’s act of generosity “of charity, gratitude, fidelity, generosity or any other strong display of virtue” (Algoe & Haidt, 2009). The constellation of emotional, physical and psychological aspects of a Moral Elevation response have been well described (see Haidt, 2003). Moral Elevation can be elicited with stimulus stories or videos (Algoe & Haidt, 2009) and measured with self-report questionnaires (Aquino et al., 2011; Freeman et al., 2009). The experience of Moral Elevation is associated with subsequent prosocial and affiliative behaviors (Algoe & Haidt, 2009). And in our prior work we have linked Moral Elevation response with behavior on the AlAn’s game (Sakai et al., 2016). In this study we will test whether behavior on the AlAn’s Short Game v.2 is related to Moral Elevation response and extend our prior work by testing whether viewing the Moral Elevation stimulus prior to playing the AlAn’s Short Game v.2 (vs. viewing a control nature video) is associated with differences in game behavior (hypothesis 2). We will also test whether psychopathic traits will correlate with game behavior (hypothesis 3 and 4).

**III**. **Preliminary Studies/Progress Report:**

We utilized the first version of the AlAn’s Short Game in a previous study with young adults. We also previously studied adolescent patient and typically developing populations using the AlAn’s game (see Sakai et al., 2012; 2016; under COMIRB #’s 07-0213 & 12-0117).

**IV. Research** **Methods**

**A. Outcome Measure(s):**

Outcome measures will be based on responses to the online survey and game administered through REDCap. Outcomes include examining if the number of accepted trials of two active trial types: 1) differ for those subjects who watched the Elevation vs. Nature video before playing the AlAn’s Short Game v.2 or 2) significantly correlate with psychopathic trait scores as measured on the Levenson Self Report Psychopathy Scale. Secondary outcomes are levels of social connectedness.

1. **Description of Population to be Enrolled:**

We will recruit up to 720 participants, 120 already tested on the first version of the AlAn’s Game, and then an additional 500 emerging adults (18-25 years of age) from the community on the AlAn’s Short Game v.2 to attain 200 subjects with useable data in each of the two study arms. Subjects may be recruited through flyers, advertisements (i.e. Craigslist), or a research marketing company. We seek to enroll a community-based sample. Therefore our inclusion/exclusion criteria are minimal.

Inclusion criteria: (1) age 18-25 years.

Exclusion criterion: (1) refusal to provide informed consent.

1. **Study Design and Research Methods**

Recruitment procedures:

Individuals who express an interest in participating in the research study (e.g., by responding to an advertisement or through contact with a research marketing company), will be contacted by the Research Coordinator or other trained PRA, who will verify subjects’ identity/age and briefly describe the study to ascertain interest.

Consent procedures:

For all subjects we will follow the standard recommended procedures to obtain online consent through REDCap. Subjects who do not have computer or email access will be given the opportunity to complete the same procedures listed below on a researcher’s computer. The Postcard Consent Information Sheet acts as the consent form and consent process for this study. If a subject agrees to participate, they will be given a link to the online consent and a unique token number. The token number will be their subject number and will allow them access to the online Postcard Consent Information Sheet upon following the provided link.

The Postcard Consent Information Sheet is presented to the subject first, and is separated from the online survey. Potential participants will be required to click a box at the end of the consent form that says, “Yes, I agree to be in this study” prior to being granted access to the survey. Only those subjects who click this box will be able to complete the survey. In addition, the contact number for the PI and Research Coordinator are provided on the Postcard Consent Information Sheet and subjects are encouraged to contact them with any questions.

The online Postcard Consent Information Sheet will collect each subject’s first and last name, email address and the date of consent. The Survey will then be presented in a separate online document and those responses will be identified only by the unique token/subject number and will not contain any PHI or other identifying information. Completed Postcard Consent Information Sheets and survey responses are securely stored separately from each other. Only PI approved research study personnel with a legitimate business purpose will have access to PHI, token/subject numbers and research data. All research data will be stored within the REDCap system or on the secure University server.

*A Waiver of Documentation of Consent and HIPAA Authorization will allow us to obtain online subject consent thru REDCap.*

*Justification for Documentation of Consent and Authorization Waiver:*

To maximize subject participation we utilize online consent and survey collection. Without such procedures in place it would not be feasible to collect a large enough sample to accomplish the aims of this project. We are not able to meet in person with all participants to conduct a fully valid written consent process. This minimal risk research project could not practicably be carried out without this waiver.

Participants will consent to the study and then be assigned to one of the two study arms. This study involves subjects completing the 35-45 minute online study via REDCap one time. Subjects will provide demographic information, complete three questionnaires, play the AlAn’s Short Game v.2 twice, and view either a short Moral Elevation stimulus or control video. (See Figure 1 below.)

The REDCap system provides access to electronic copies of completed consent forms as documentation that consent was obtained for every subject who completes the online survey. Subjects will receive an electronic PDF copy of their completed Postcard Consent Information Sheet.

This project presents no more than minimal risk of harm to subjects. The probability and magnitude of physical or psychological harm or discomfort involved in this research project are not greater in and of themselves than those ordinarily encountered in daily life or during the performance of routine physical or psychological examinations or tests. The PI and research staff will rigorously monitor this research project to ensure safety of all research subjects. They will protect the privacy and confidentiality of research subjects.

Compensation:

Upon completion of the online survey participants will be paid $20 with an electronic gift card (plus whatever the subject earned while playing the AlAn’s Short Game v.2, up to an additional $0.30 - $9.70). If the participant does not watch the videos in the project in their entirety then their payment will be reduced by $10. A timestamp within the REDCap program will accurately track this and the subject is reminded of this before watching each video. The Research Study Coordinator will buy the electronic gift cards using her University procurement card and a link to the gift card will be delivered to the participant. All University procedures related to documentation of study subject payments and the use of electronic gift cards will be followed.

**D. Description, Risks and Justification of Procedures and Data Collection Tools:**

Data Collection Tools:

The online survey is designed to build upon previous research of prosocial behavior and associated phenotypes in a young adult population. In REDCap, subjects play the AlAn’s Short Game v.2, view the Moral Elevation Stimulus or Control Stimulus Video and complete questionnaires relevant to (1) psychopathic traits, (2) adverse childhood experiences, (3) social connectedness, and (4) demographics.

Description of AlAn’s Short Game v.2: In the AlAn’s Short Game v.2 subjects start with $3 and a real donation to the Red Cross starts at $3. Like the original AlAn’s game, there are three trial types: Active Trials, Calculation Trials and Attention Control Trials.

*Active Trials* ask “Change both counters?” and present a “You Get” amount and a separate “Red Cross” amount (see example figure below). Subjects are asked to accept vs. reject the offer and then are shown the counters which track the amount of money they have accrued and the amount remaining in the Red Cross donation. This shortened game includes 22 Active Trials (11 of each type), which are each presented to the subject. Those trials include these pairs (see Table to right). Active Trials Type 1 (AT1) include trials where Red Cross donation will be reduced, and the subject will gain money. Active Trials Type 2 (AT2) will include trials where the Red Cross donation will increase and the participant will lose money.

| Active Trials in Alan’s Short Game v.2 | |
| --- | --- |
| AT1 | AT2 |
| you get 32, RC loses 4 | you lose  24, RC gets 6 |
| you get 4, RC loses 32 | you lose 16, RC gets 8 |
| you get 12, RC loses 12 | you lose 48, RC gets 3 |
| you get 48, RC loses 3 | you lose 6, RC gets 24 |
| you get 6, RC loses 24 | you lose 4, RC gets 32 |
| you get 64, RC loses 2 | you lose 32, RC gets 4 |
| you get 3, RC loses 48 | you lose 3, RC gets 48 |
| you get 8, RC loses 16 | you lose 8, RC gets 16 |
| you get 24, RC loses 6 | you lose 2, RC gets 64 |
| you get 16, RC loses 8 | you lose 64, RC gets 2 |
| you get 2, RC loses 64 | you lose 12, RC gets 12 |


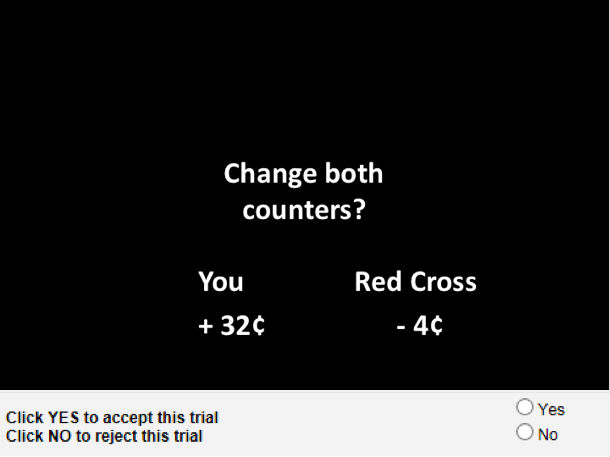

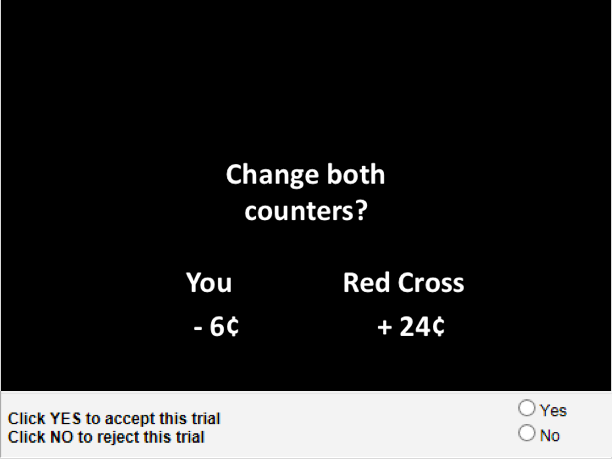
 Active Trial 1 (AT1): Active Trial 2 (AT2):

*Calculation Trials* ask subjects to determine “Is the You number bigger?” If the You number is bigger than the subject should click “Yes”. If the You number is not bigger, they should answer “No”. The same You and Red Cross numbers are used as in the Active Trials (except the 12,12 where you and the Red Cross are equal). Therefore there are 10 Calculation Trials in the AlAn’s Short Game v.2. These trials allow objectively testing subject’s performance in understanding these relative values and serves as a check on attention to the task.

Calculation Trial:

*
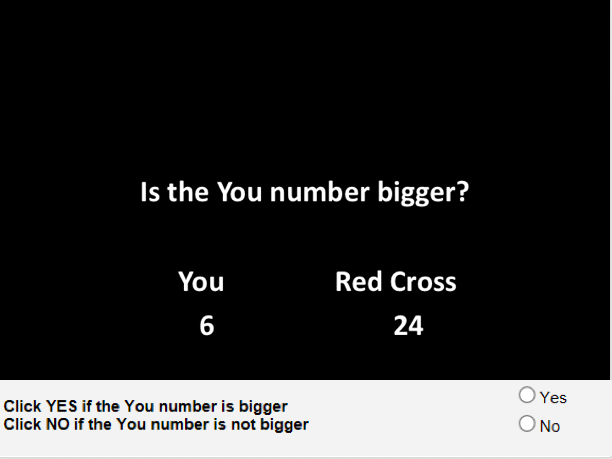
*

*Attention Control Trials.*  Subjects are presented with 2 trials that they should logically accept (where both they and the Red Cross will earn money) and 2 logically reject trials (where both they and the Red Cross will lose money). This keeps subjects from simply falling into a pattern of always accepting (or always rejecting) Active Trials. Examples of each type are shown below.

Logically Reject Trial: Logically Accept Trial:


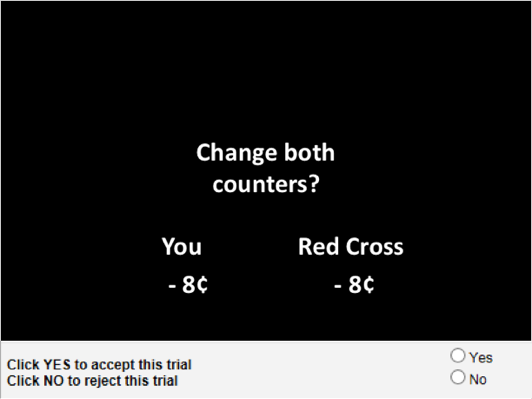

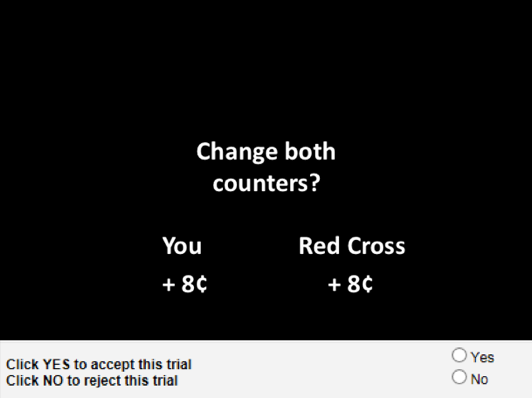


Moral Elevation Stimulus: Participants will view a short video about either an uncommon act of selflessness, that has been previously used as a stimulus to elicit Moral Elevation (Englander et al., 2012; <http://www.youtube.com/watch?v=e9JcX2X7XnM>) or a Control Nature Video at <https://www.youtube.com/watch?v=9Qe1uL9Tzg8>. Subjects will then play AlAn’s Short Game v.2 a second time.

Measures of psychopathic traits and externalizing behavior: Subjects will complete the *Levenson Self-Report Psychopathy Scale* (Levenson et al., 1995). The Levenson provides a factor 1 and factor 2 score (which is related to externalizing behavior).

Social Connectedness will be measured by the *relatedness subscale of the Basic Psychological Needs Scale* (Basic Need Satisfaction in General; Deci & Ryan, 2000) to provide a measure of connectedness to others.

Adverse childhood experiences: Subjects will complete the Adverse Childhood Events (ACES) questionnaire (Edwards et al., 2003).

Demographics: Gender, age and race/ethnicity.

Order of Administration: We will randomly assign subjects to receive one of two possible orders of administration (as described in Figure 1 below). Arm 1 presents the Moral Elevation stimulus after playing the first AlAn’s Short Game v.2 (session 1), while Arm 2 presents the control stimulus (nature video). Subjects in both arms will then play AlAn’s game again. After playing the game for a second time, subjects in both arms will fill out an Adverse Childhood Experiences Questionnaire (ACES), Social Connectedness survey, and Demographics form.

Figure 1: Study Arms and Order of Administration


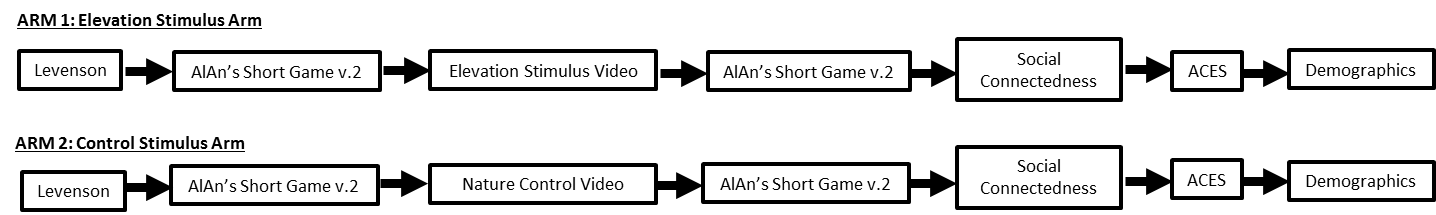


Data Management:

Study data will be collected and managed using REDCap (Research Electronic Data Capture, Harris et al 2008). REDCap is a secure web application designed to support data capture for research studies, providing user-friendly web-based case report forms, real-time data entry validation (e.g. for data types and range checks), audit trails and a de-identified data export mechanism to common statistical packages (SPSS, SAS, Stata, R/S-Plus). The system was developed by a multi-institutional consortium which includes University of Colorado–Denver and was initiated at Vanderbilt University. The database is hosted at the University of Colorado–Denver Development and Informatics Service Center (DISC), which will be used as a central location for data processing and management. REDCap data collection projects rely on a thorough study-specific data dictionary defined in an iterative self-documenting process by all members of the research team with planning assistance from the *DISC*. This iterative development and testing process results in a well-planned data collection strategy for individual studies. REDCap also includes a powerful tool for building and managing online surveys. The research team can create and design surveys in a web browser and engage potential respondents using a variety of notification methods. REDCap is flexible enough to be used for a variety of types of research and provides an intuitive user interface for database and survey design and data entry.

Risks:

1) The major risk to subjects participating in this research study is a breach of confidentiality. Subjects will play a game of prosocial behavior and describe adverse childhood experiences. We will also talk about this research study at meetings. We might also print the results of this research study in relevant journals or share data that does not identify subjects with qualified investigators or as a supplement as required by some journals. However, the names and any other identifying information of research subjects will always be kept confidential.

2) Subjects may experience some embarrassment or nervous tension during completion of the online survey.

Protection Against Risks:

*1) Breaches of Confidentiality*

Our best defense against breaches of confidentiality is the strong and careful attention paid to this issue by the senior investigator and experienced research staff. The PI and research staff will rigorously monitor this research project to ensure safety of all research subjects. They will protect the privacy and confidentiality of research subjects. The PI and his research team are highly experienced in protecting the confidentiality of these subjects. All research staff are kept fully aware of the importance of protecting confidentiality, and are trained according to COMIRB’s current standards.

Our second defense is that only subject code numbers and not names will identify individuals in the online survey. Only authorized research personnel with a legitimate business purpose will have access to the code numbers. The online consent form, which will contain each subject’s full name, will be stored in a separately in the REDCap database. We will utilize a function within the REDCap application that allows records to be “locked” so that only authorized personnel with a legitimate business purpose will have access to these records.

The third defense against breaches of confidentiality is that all research materials will be securely stored at all times. All data collected in REDCap will be stored on secure University servers. Access to data is restricted to authorized research personnel with a legitimate business purpose. To ensure that REDCap users have access only to their own data and information, user privileges are utilized within the software. Each user has their own account, and their user account will only have access to REDCap projects that they themselves have created or to projects to which other users have granted them access. REDCap also has a built-in audit trail that automatically logs all user activity and logs all pages viewed by every user, including contextual information (e.g. the project or record being accessed). To help protect and secure the data stored in REDCap’s back end database, the software application employs various methods to protect against malicious users who may attempt to identify and exploit any security vulnerabilities in the system. Both REDCap and REDCap Survey completed an extensive 6-week internet security threat assessment evaluation by Tim McCain, former UCD HIPAA Security Officer. Both systems were found to meet or exceed all of the security audit requirements. See http://www.project-redcap.org/ for technical details and more information.

*2) Embarrassment or Nervous Tension*

The survey will be self-administered online, minimizing embarrassment or nervous tension. In addition, subjects will be able to stop the survey at any time.

This project presents no more than minimal risk of harm to subjects. The probability and magnitude of physical or psychological harm or discomfort involved in this research project are not greater in and of themselves than those ordinarily encountered in daily life or during the performance of routine physical or psychological examinations or tests. The risks to individual subjects are quite small. The risk/benefit ratio is favorable.

**E. Potential Scientific Problems:**

Recruitment is always a potential scientific problem. Without the appropriate participants, the results may not be valid. We will devote the necessary resources to contact an adequate number of participants and ask them to consent and complete the survey online. In addition, the Waiver of Documentation of Consent and HIPAA Authorization will be highly beneficial and will allow this study to collect the data needed to accomplish the aims of this project and yield preliminary data for future grant applications.

**F. Data Analysis Plan:**

Brief description of planned analyses:

We will collect 400 subjects with useable data; 200 who watch the elevation stimulus (Order of Administration 1) and 200 who watch the control video (Order of Administration 2).

**Aim 1.** Similar to our prior work we will start by visually inspecting our data. We will graph Active Trial ratio (X-Axis; ratio=absolute value of change to Red Cross donation divided by You gain amounts) for Arm 1 and 2 and sessions 1 and 2 of the AlAn’s Short Game v.2. We have previously used this graphing approach in our prior work, where we exclusively included AT1. But we have not previously included Active Trials with negative you amounts and positive Red Cross amounts (e.g., you lose 2 cents and the Red Cross donation goes up by 64 cents; AT2). Those trials will provide negative ratios on the X-axis and we expect the average pattern of response to be similar to those seen in the AT1 trials (double mirror image – reflecting in both the horizontal and vertical direction - presented in the associated figure). For example, we would expect the ratios where the Red Cross gain amount is very large and the you lose amount is small [(|64|/-2)=ratio of -32] to be accepted at a high rate. But as the negative ratios approach zero (e.g., Red Cross gain 2 cents, you lose 64 cents = ratio of -0.03125) the acceptance rates would decline. It is possible that the acceptance rate curve will be shifted downward in comparison to expectation due to effects of loss aversion. Next we will graph number of yes responses to AT1 trials against AT2 trials for session 1 across all subjects and calculate correlations (Spearman if our data do not approximate normality).

Note: If AT1 and AT2 significantly correlate under Aim 1, we will use outcomes (costly helping, amount of money taken for self, amount of money left in the donation) from all Active Trials. If AT1 and AT2 do not significantly correlate, we will use only AT1 trials in subsequent analyses (e.g., Aims 2-3).

**Aim 2.** Our expectation is that both groups will show similar behavior during session 1, but that in session 2, prosocial behavior will increase for subjects in Arm 1 but not Arm 2. We will again begin by graphing our data. First we will graph session 1 and 2 AlAn’s Short Game v.2 behavior (costly helping, money taken for self, money left in the donation) for each session within Arm. We will also graph session 1 vs. session 2 results. In prior work with the original AlAn’s game, session 1 and 2 results (separated by a short period of watching a view of the ocean) significantly correlated (money taken for self rho=0.82 and money left in the donation rho=0.91). We would expect that to be the case again for Arm 2 (where subjects watch a nature video). After visually inspecting our results, we will then complete a two-way repeated measures ANOVA examining study arm by session.

**Aim 3.** We will start by correlating psychopathic trait scores with AlAn’s Short Game v.2 behavior from session 1 (all subjects). We expect that as psychopathic trait scores increase that prosocial behavior on the game will decrease. Next we will graph change in prosocial behavior (session 2 minus session 1) and level of psychopathic traits for subjects in Arm 1. We expect a negative relationship between these two variables and predict that the effects of Elevation will be less prominent in those with high psychopathic traits.

**Power Analyses and Sample Size Estimation:**

In most proposed analyses, we will be completing correlation analyses. In some instances, we will restrict our sample to those in Arm 1. With a sample size of 150 subjects in Arm 1, we expect to be able to detect correlations where r≥0.25 (assuming α=0.05 and β=0.20). Correlations of lower value than this are likely not a clinical significance. Our least powered analyses are under Aim 2, which examine within subject change between two groups. We have preliminary data collected on subjects who played the AlAn’s Short Game after vs. before watching the Elevation stimulus. Although imperfect, these data provide expected group differences in mean (Costly Helping 1.4, Red Cross donation 0.14 and money taken for self -0.44) and standard deviation (6.3, 1.43, 1.3, respectively). We calculate, assuming α=0.05 and β=0.20, that we need samples sizes of about 200 per group on the AlAn’s Short Game v.2.

**G. Summarize Knowledge to be Gained:**

We have previously developed a behavioral measure of costly helping and prosocial behavior called the AlAn’s game and then we developed the online AlAn’s Short Game. In this proposal, we seek to test the validity of the AlAn’s Short Game v.2 which can also be delivered through the REDCap platform. If behavior on this second version of the game can be related to levels of psychopathic traits it would provide a measure that is much more easily exported to other labs and which can be efficiently utilized to answer a broad range of research questions. The proposed work on the second version of the AlAn’s Short Game will further extend our prior work on the link between Moral Elevation response and prosocial behaviors. If Moral Elevation response is related to increased levels of prosocial behavior, this will extend our understanding of mechanisms which may underlie psychopathic traits.

**H. References:**

Algoe SB, Haidt J. Witnessing excellence in action: the 'other-praising' emotions of elevation, gratitude, and admiration. J Posit Psychol. 2009;4(2):105-27. doi: 10.1080/17439760802650519.

Aquino K, McFerran B, Laven M. Moral identity and the experience of moral elevation in response to acts of uncommon goodness. J Pers Soc Psychol. 2011;100(4):703-18. doi: 10.1037/a0022540.

Deci EL, Ryan RM. The “what” and “why” of goal pursuits: Human needs and the self determination of behavior. Psychological Inquiry. 2000; 11:227-268.

Edwards VJ, Holden GW, Anda RF, Felitti VJ. [Relationship between multiple forms of childhood maltreatment and adult mental health in community respondents: results from the Adverse Childhood Experiences (ACE) Study](http://www.ncbi.nlm.nih.gov/entrez/query.fcgi?cmd=Retrieve&db=pubmed&dopt=Abstract&list_uids=12900308). American Journal of Psychiatry 2003;160(8):1453–60.

Englander ZA, Haidt J, Morris JP. Neural basis of moral elevation demonstrated through inter-subject synchronization of cortical activity during free-viewing. PLoS One. 2012;7(6):e39384. doi: 10.1371/journal.pone.0039384.

Freeman D, Aquino K, McFerran B. Overcoming beneficiary race as an impediment to charitable donations: social dominance orientation, the experience of moral elevation, and donation behavior. Pers Soc Psychol Bull. 2009;35(1):72-84. doi: 10.1177/0146167208325415.

Frick PJ. (2004). The Inventory of Callous-Unemotional traits. New Orleans, LA: University of Louisiana.

Haidt J. Elevation and the positive psychology of morality. In: Keyes C, Haidt, J., editor. Flourishing: Positive psychology and the life well-lived. Washington, DC: American Psychological Association; 2003. p. 275-89

Harris PA, Thielke R, Taylor R, Payne J, Gonzalez N, Conde JG. (2008). Research Electronic Data Capture (REDCap) - A metadata-driven methodology and workflow process for providing translational research informatics support. Journal of Biomedical Informatics.

Levenson MR, Kiehl KA, Fitzpatrick CM. Assessing psychopathic attributes in a noninstitutionalized population. Journal of Personality and Social Psychology. 1995; 68:151-8.

Sakai JT, Dalwani MS, Mikulich-Gilbertson SK, McWilliams SK, Raymond KM, Crowley TJ. A Behavioral Measure of Costly Helping: Replicating and Extending the Association with Callous Unemotional Traits in Male Adolescents. PLoS One. 2016;11(3):e0151678. doi: 10.1371/journal.pone.0151678.

Sakai JT, Dalwani MS, Gelhorn HL, Mikulich-Gilbertson SK, Crowley TJ. A behavioral test of accepting benefits that cost others: associations with conduct problems and callous-unemotionality. PLoS One. 2012;7(4):e36158. doi: 10.1371/journal.pone.0036158.

Sakai JT, Dalwani MS, Mikulich-Gilbertson SK, Raymond K, McWilliams S, Tanabe J, Rojas D, Regner M, Banich MT, Crowley TJ. Imaging Decision about Whether to, or Not to, Benefit Self by Harming Others: Adolescents with Conduct and Substance Problems, with or without Callous-Unemotionality, or Developing Typically. Psychiatry Research Neuroimaging (under review – in revision).
